# High-throughput Method of One-Step DNA Isolation for PCR Diagnostics of Mycobacterium tuberculosis

**Published:** 2014

**Authors:** D. V. Kapustin, A. I. Prostyakova, Ya. I. Alexeev, D. A. Varlamov, V. P. Zubov, S. K. Zavriev

**Affiliations:** M.M. Shemyakin and Yu.A. Ovchinnikov Institute of Bioorganic Chemistry of the Russian Academy of Sciences, GSP-7, Ul. Miklukho-Maklaya, 16/10, Moscow, Russia, 117997; ZAO SINTOL, Ul. Timiryazevskaya, 42, Moscow, Russia, 127550; Russian National Research Institute of Agricultural Biotechnology of the Russian Academy of Agricultural Sciences, Ul. Timiryazevskaya, 42, Moscow, Russia, 127550

**Keywords:** composite sorbents, DNA isolation, PCR diagnostics, Mycobacterium tuberculosis complex, fluoropolymers, polyaniline

## Abstract

The efficiency of one-step and multi-step protocols of DNA isolation from lysed sputum samples containing the Mycobacterium tuberculosis complex has been compared. DNA was isolated using spin-cartridges containing a special silica-based sorbent modified with fluoroplast and polyaniline, or using an automated isolation system. One-step isolation using the obtained sorbent has been shown to ensure a significantly lower DNA loss and higher sensitivity in the PCR detection of Mycobacterium tuberculosis as compared to a system based on sorption and desorption of nucleic acids during the isolation.

## INTRODUCTION

Purified preparations of nucleic acids isolated from different biological sources are increasingly used in biomedical studies (e.g., in sequencing, as immunomodulating or anticancer agents, in designing drug delivery vehicles, etc.), especially in medical diagnosis and bioassay, largely owing to the successful development of PCR technology. The efficiency of sample preparation techniques depends on the development of rapid and reproducible methods for the isolation of DNA that is readily suitable for PCR diagnostics. Various methods for nucleic acid isolation have been described; commercial kits suitable for isolation are available and being used both to solve research problems and in clinical practice. The use of silica particles for DNA sorption in the presence of chaotropic salt was first proposed as early as 1979 [1]. In 1990, the method was improved [2] and some variations are used to this day. Nucleic acid isolation methods using magnetic particles based on silica [3], fibers modified with silica particles [4], affine silica particles [5], etc., are widely used.

Methods of biopolymers separation and isolation are typically based on the differences in the solubilities of nucleic acids, proteins, and polysaccharides. These methods are based on “capturing” the target biopolymer from a mixture with a sorbent and retaining it during the first stage of separation, removing impurities, and eluting the target component from the sorbent surface during the following stages. Thus, the isolation procedures are multi-step, laborious, time-consuming, difficult to automate, and do not always ensure sufficient purity of the isolated DNA for effective PCR assay. The latter generally depends on the presence of PCR inhibitors in the sample (e.g., heme in blood samples, various types of chlorophyll in plant tissue lysates, humic acids in soil samples, etc.).

The one-step extraction scheme is obviously more attractive and promising. In this case, the isolated (and simultaneously purified) biopolymer passes through the sorbent layer without retention on its surface, while the impurities hindering PCR are effectively retained by the sorbent. Several years ago, we developed a onestep procedure for DNA isolation which is based on using the unique sorption properties of certain polymers, namely fluoropolymer (FP) [6] and polyaniline (PANI) [7, 8]. Such polymer coatings formed, for example, on the porous silica surface do not retain DNA but adsorb the proteins and PCR -inhibitory components that are present in typical biological samples (plant or bacterial lysates, smears, plasma, blood, etc.).

We have developed several methods for synthesizing fluoropolymer- and PANI-containing composite sorbents based on various physical and chemical processes [9, 10]. Both the fluoropolymers and PANI being used as surface modifiers of sorbents make possible onestep isolation of nucleic acids from complex biological mixtures. However, each of these polymeric modifiers brings additional significant useful properties. Thus, fluoropolymer-containing sorbents are noted for exceptional chemical resistance, low nonspecific adsorption, and usually they provide the highest yield of DNA. In turn, the PANI-containing sorbents that have excellent wettability and high surface capacity can be successfully used to isolate DNA from “complex” biological mixtures (blood, plant tissue lysates, soil extracts, etc.), as well as to separate nucleic acids depending on their secondary structure.

In this regard, materials modified with both fluoropolymer and PANI nanolayers simultaneously are of particular interest. We have reported the successful application of such material for the one-step isolation of predominantly double-stranded DNA of the human hepatitis B virus and single-stranded DNA of the TT V virus (*Transfusion transmitted virus, Torque teno virus* is the virus that is transmitted during blood transfusion) suitable for PCR assay from human plasma samples [11]. However, the applicability of these sorbents for rapid extraction of human non-viral pathogen DNA from clinical samples remains insufficiently studied. This article discusses the use of a silica sorbent modified with a combination of FP and PANI nanolayers for one-step DNA extraction from inactivated clinical sputum samples containing different amounts of cells of human tuberculosis pathogens known under the common name “*Mycobacterium tuberculosis complex*” (MTC ) and including the following mycobacterial species:* M. tuberculosis, M. bovis, M. bovis BCG, M. africanum, M. microti, M. canettii, M. caprae, M. pinnipedii,* and *M. mungi*.

The efficiency of one-step DNA extraction using cartridges containing the FP-PANI sorbent was compared with the automated multi-step method of isolation of DNA from MTC that was developed by the ZAO Syntol company (Moscow, Russia) based on a Tecan Freedom EVO ® PCR robotic station (Tecan Trading AG, Switzerland).

## EXPERIMENTAL

Aniline (extra-pure grade, Aldrich, Germany) was distilled, and a fraction with n^20^_d_ 1.5863 was collected in a temperature range of 182–184°C. Ammonium persulfate (Rotipuran ®, Germany), hydrochloric acid, ethanol (reagent grade, Aldrich, Germany), and water (Milli Q standard) were used.

DNA of tuberculosis mycobacteria was isolated from sputum samples using plastic spin cartridges containing 100 mg of the Si-500-FP-PANI sorbent and a Tecan Freedom EVO ® PCR automated robotic station (Tecan Trading AG, Switzerland) that was adapted to the M-Sorb-Tub-Avtomat kit (ZAO Syntol, Russia) used for automated DNA isolation. Sputum samples were lysed using reagents supplied with the AmpliTub-RV kit (ZAO Syntol, Russia), intended for qualitative and quantitative determination of *Mycobacterium tuberculosis complex *using real-time PCR (lysis reagent B+, diluent, inactivating reagent A).

In this work, we used the following additional equipment: the Tsiklotemp-903 centrifuge for microtubes; a Tsiklotemp-303 thermostat; a Tsiklotemp-901 microcentrifuge shaker for microtubes (all equipment manufactured by ZAO Tsiklotemp, Russia); 20, 200, and 1000 µl variable volume micropipettes; a 1.5-ml test tube holder; 1.5 ml microtubes; and micropipette tips.

**Preparation of the FP-PANI sorbent**

Si-500 silica (100 g) was vacuumized in a special reactor for 30–40 min, and then 500 ml of a 0.016% FP solution in acetone was added to the silica. The reactor containing the suspension of silica particles was incubated in an ultrasonic bath at atmospheric pressure for 15 min. The solvent was then removed on a rotary evaporator at 40–45°C. Another portion of the polymer solution in acetone (500 ml) was injected into the reactor, and the manipulations following the injection of the first portion of the polymer solution were repeated. The resulting product was dried in vacuo to a constant weight and used as a matrix during the oxidative polymerization of aniline as described in [11].

**Protocol for isolation of mycobacterial DNA using cartridges containing the FP-PANI sorbent**

*Inactivation*. Model and clinical sputum samples were used in the experiments. 500 µl of the inactivating reagent A was added to 500 µl of sputum containing 600 CFU of MTC /ml (in particular, *M. tuberculosis, M. bovis, M. bovis BCG*, etc.) to inactivate model samples. All clinical samples were pre-inactivated as well: the inactivating reagent A was added to the sputum samples collected in 50-ml test tubes to a final volume of 40 ml. The contents of the test tube were mixed by gently turning the test tube over until complete homogenization, and then they were incubated for 30 min. The tubes were centrifuged (15 min at 3500 rpm). The supernatant was discarded; the precipitate was re-suspended and transferred into 1.5-ml tubes.

*Lysis*. The tubes with the test material were centrifuged (5 min at 13,000 rpm); the supernatant was discarded, and 100 µl of the lysis reagent B+ was added. The contents of the tubes were thoroughly mixed in the microcentrifuge shaker and incubated for 10 min at 75°C. The tubes were centrifuged in the microcentrifuge for 15 seconds. The DNA obtained was used for PCR using AmpliTub-RV (for manual DNA extraction) and M-Sorb-Tub-Avtomat (for automated DNA extraction) reagent kits for the detection of a Mycobacterium tuberculosis complex.

*DNA purification*. 400 µl of the diluent was added to the sample tubes and stirred. The contents of the tubes were applied by gentle pipetting on the sorbent cartridges that were inserted into the collector tubes. The cartridges were centrifuged for 1 min at 4,000 rpm and then removed from the collectors.

*Real-time PCR*. Real-time PCR was performed using an ANK-32 analyzer (Institute for Analytical Instrumentation of the Russian Academy of Sciences, Russia) and the appropriate algorithm developed by ZAO Syntol. The diagnostics method used allows one to detect the presence of specific DNA fragments from the IS6110 gene in the sample, which is present in multiple copies in most MTC strains, but may be absent in the genome of *M. tuberculosis*. At the same time, the method allows one to determine the amount of *regX, *a specific DNA fragment which is represented by a single copy in the MTC genome.

Conditions for the PCR using the isolated DNA were identical.

## RESULTS AND DISCUSSION

Experiments aimed at isolating mycobacterial DNA from a control model and clinical sputum samples were performed at ZAO Syntol (Moscow, Russia). We compared the efficiency of the two methods of DNA isolation from lysed sputum samples: using a FP-PANI sorbent containing cartridges, and using the automated isolation system produced on the basis of Tecan Freedom EVO ® PCR robotic station (Tecan Trading AG, Switzerland). In the latter case, the procedure of DNA isolation was based on the adsorption of the nucleic acid molecules of a tuberculosis pathogen on oligonucleotide- coated magnetic particles. The nucleotide sequence of these oligonucleotides was complementary to the target sequence in the pathogenic DNA. As a result, the pathogenic DNA binds complementarily to the matrix surface under the conditions provided by the system. The impurities not bound to the magnetic particles are then removed automatically (including DNA that does not contain the target sequence), and the purified DNA is eluted from the surface of the magnetic particles. In such a way, the multi-step process of DNA isolation is implemented in the automated system. On the contrary, one-step isolation of bacterial DNA takes place when the FP-PANI sorbent is used. The isolated DNA samples were analyzed by real-time PCR . In this way, the efficiency of the amplification of PCR fragments of DNA was assessed after one-step isolation using the FP-PANI sorbent and after multi-step automated isolation. Model sputum samples containing 600 CFU/ml and clinical samples obtained from randomly selected patients were investigated.

**Table 1:** Threshold cycle values and the calculated number of DNA copies in the model samples after real-time PCR

Sample	Threshold cycle, Ct	Calculated number of DNA copies
Original lysate	40.2	0.4
Original lysate	41.03	0.22
Diluted lysate	35.23	13.72
1	35.28	13.19
2	34.27	27.10
3	33.77	38.50
4	33.70	40.41
5	35.69	9.84
6	35.28	13.19
7	35.04	15.69
8	34.66	20.48
9	35.08	15.24
Control dilutions		
10,000,000	16.20	9.948E6
100,000	21.58	2.193E5
1000	29.14	1026.34
100	32.45	97.94

*Note*. 1–9 – The model sputum samples containing 600 CFU/ml after passing through the cartridge containing the FP-PANI sorbent.

The results of model samples testing are shown in the *Figure* and *Table. 1*. The *Figure* shows that the efficiency of the amplification of the PCR DNA fragments that were not subjected to further purification on the cartridges with the sorbent (i.e., the original unpurified lysates) was significantly lower (curves shown by an arrow in *Fig. A*, “original lysate” sample in *Table 1*). However, the efficiency of the amplification is improved after dilution of these samples (due to the reduction in the relative concentration of PCR inhibitors in the test sample), and the number of PCR fragments becomes comparable to that of the amplicons obtained using the DNA samples that were purified using the FP-PANI sorbent (*Fig. B*, “diluted lysate” sample in *Table. 1*). The control values of the threshold cycle and the relative concentrations of DNA in the samples with a known amount of DNA (107, 105, 103, and 102 CFU, respectively) are also shown in *Table 1*. It can be seen that the use of the FP-PANI sorbent does not reduce the sensitivity of PCR detection and allows one to identify about 10 copies of the analyzed DNA in the sample. Therefore, the FP-PANI sorbent provides effective removal of PCR inhibitors and maintains the original amount of DNA in the test sample. Based on these data, we can assume that FP-PANI-containing material is efficient for one-step isolation of DNA from clinical samples and provides purified preparations of nucleic acids that are suitable for PCR analysis.

**Fig. 1:**
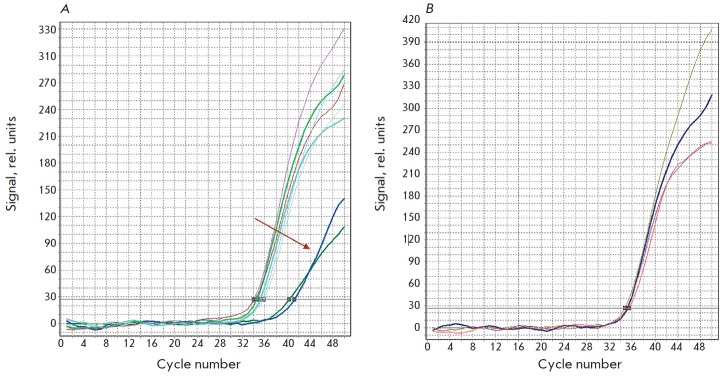
The results of real-time PCR of the mycobacterial DNA isolated from the model sputum lysates using the FP-PANI sorbent and containing 600 CFU/ml. A – the arrow points to the curves obtained using samples that were not purified on the sorbent (original lysates); B – red and brown curves –samples purified using the FP-PANI sorbent; blue curve – the original lysate sample after dilution without further purification using the FP-PANI sorbent

The amounts of DNA extracted from clinical samples using a FP-PANI sorbent and an automated system were compared to confirm this assumption. The amount of starting material for the automated system was 2 times higher than the amount taken for the isolation on cartridges. However, given that only half of the volume taken is used during the automated isolation, the amount of starting material is comparable in both cases. When using the automated system, the final volume of the DNA solution was fourfold lower, and the amount required for PCR was 2.5 times higher than that in the case of DNA isolation on cartridges. Thus, the amount of DNA for PCR in the automated system is 10 times higher than the amount of DNA for PCR after manual isolation using cartridges.

*Table 2* shows the number of PCR fragments of DNA from mycobacteria obtained when analyzing clinical samples by both methods and the amount of DNA determined by automated amplification with allowance for dilution.

**Table 2:** Number of PCR fragments of DNA from the *M. tuberculosis complex *after the cartridge and automated isolation of DNA from the clinical samples

Sample	Number of DNA, copies/volume		
	Cartridges, 10 µl	Automated isolation, 25 µl	Automated isolation, after dilution
1	4579	3254	325
2	65	Not determined	Not determined
3	5006	3572	357
4	Not determined	Not determined	Not determined
5	733220	23693	2369
6	98	3	< 1
7	12	2	< 1
8	178	32	3

It follows from the results shown in *Table 2* that the efficiency of automated DNA extraction was 3 to 7% comparable to the extraction using the FP-PANI sorbent.

Therefore, the use of a sorbent modified with a combination of FP and PANI nanolayers substantially reduces DNA loss and provides for a much more sensitive detection of *M. tuberculosis *DNA as compared to the system based on absorption and desorption of nucleic acids during the isolation.

## CONCLUSIONS

The methods for isolating nucleic acids from biological mixtures usually involve three different physicochemical processes: extraction, precipitation or adsorption of the target component (nucleic acid) on the sorbent surface, followed by washing of impurities and desorption. These procedures are laborious and often result in a significant loss of the nucleic acid being isolated.

The one-step scheme of nucleic acid isolation using special sorbents seems to be an efficient alternative to multi-step protocols. In this method, the nucleic acid is not retained by the sorbent, while the impurities contained in the original mixture (especially PCR inhibitors) are firmly adsorbed. Due to the unique sorption properties of some synthetic polymers, such as fluoropolymers and PANI, it was possible to develop several composite sorbents that provide one-step isolation of nucleic acids from complex biological mixtures. These sorbents are characterized by a high selectivity when separating nucleic acids and proteins. It is of particular interest to study the sorption properties of a sorbent sequentially modified with FP and PANI nanolayers. This material combines high chemical resistance due to the presence of a fluoropolymer coating and high sorption capacity, determined by the properties of PANI coating.

This paper demonstrates that a sorbent modified with a combination of FP and PANI nanolayers efficiently removes PCR inhibitors and preserves the initial amount of DNA in the sample, as shown by the isolation of *M. tuberculosis *DNA from clinical sputum samples. Due to this fact, high sensitivity in the detection of *M. tuberculosis complex *DNA can be achieved as compared to a system based on absorption and desorption of nucleic acids during isolation.

## References

[1] Vogelstein B., Gillespie D. (1979). Proc. Natl. Acad. Sci. USA..

[2] Boom R., Sol C.J.A., Salimans M.M.M., Jansen C.L., Wertheim-van Dillen P.M.E., van der Noordaa J. (1990). J. Clin. Microbiol..

[3] Park M.E., Chang J.H. (2007). Biomimetic and Supramolecular Systems.. Mater. Sci. Eng. C..

[4] Cady N.C., Stelick S., Batt C.A. (2003). Biosens. Bioelectron..

[5] Craig J.M., Kraus J., Cremer T. (1997). Hum. Genet..

[6] Kapustin D.V., Saburov V.V., Zavada L.L., Evstratov A.V., Barsamyan G.B., Zubov V.P. (1998). Rus. J. Bioorgan. Chem..

[7] Kapustin D.V., Yagudaeva E.Yu., Zavada L.L., Zhigis L.S., Zubov V.P., Yaroshevskaya E.M., Plobner L., Leiser R.-M., Brem G. (2003). Rus. J. Bioorgan. Chem..

[8] Yagudaeva E.Yu., Muydinov M.R., Kapustin D.V., Zubov V.P. (2007). Russ. Chem. Bull. Int. Ed..

[9] Kapustin D., Prostyakova A., Bryk Ya., Yagudaeva E., Zubov V. (2011). Nanocomposites and polymers with analytical methods / Ed. Cuppoletti J. Croatia: Intech.

[10] Kapustin D.V., Prostyakova A.I., Ryazantcev D.Yu., Zubov V.P. (2011). Nanomedicine..

[11] Kapustin D., Prostyakova A., Zubov V. (2012). Zing Conferences. Polymer Chemistry Conference. 12–16 November 2012. Cancun. Mexico. Stow Cum Quy, UK,.

